# Global Profiling of DNA Replication Timing and Efficiency Reveals that Efficient Replication/Firing Occurs Late during S-Phase in *S. pombe*

**DOI:** 10.1371/journal.pone.0000722

**Published:** 2007-08-08

**Authors:** Majid Eshaghi, R. Krishna M. Karuturi, Juntao Li, Zhaoqing Chu, Edison T. Liu, Jianhua Liu

**Affiliations:** 1 Systems Biology, Genome Institute of Singapore, Singapore, Singapore; 2 Computational and Mathematical Biology, Genome Institute of Singapore, Singapore, Singapore; 3 Cancer Biology, Genome Institute of Singapore, Singapore, Singapore; 4 Department of Biochemistry, Yong Loo Lin School of Medicine, National University of Singapore, Singapore, Singapore; University of Massachusetts Medical School, United States of America

## Abstract

**Background:**

During *S. pombe* S-phase, initiation of DNA replication occurs at multiple sites (origins) that are enriched with AT-rich sequences, at various times. Current studies of genome-wide DNA replication profiles have focused on the DNA replication timing and origin location. However, the replication and/or firing efficiency of the individual origins on the genomic scale remain unclear.

**Methodology/Principal Findings:**

Using the genome-wide ORF-specific DNA microarray analysis, we show that in *S. pombe*, individual origins fire with varying efficiencies and at different times during S-phase. The increase in DNA copy number plotted as a function of time is approximated to the near-sigmoidal model, when considering the replication start and end timings at individual loci in cells released from HU-arrest. Replication efficiencies differ from origin to origin, depending on the origin's firing efficiency. We have found that DNA replication is inefficient early in S-phase, due to inefficient firing at origins. Efficient replication occurs later, attributed to efficient but late-firing origins. Furthermore, profiles of replication timing in *cds1Δ* cells are abnormal, due to the failure in resuming replication at the collapsed forks. The majority of the inefficient origins, but not the efficient ones, are found to fire in *cds1Δ* cells after HU removal, owing to the firing at the remaining unused (inefficient) origins during HU treatment.

**Conclusions/Significance:**

Taken together, our results indicate that efficient DNA replication/firing occurs late in S-phase progression in cells after HU removal, due to efficient late-firing origins. Additionally, checkpoint kinase Cds1p is required for maintaining the efficient replication/firing late in S-phase. We further propose that efficient late-firing origins are essential for ensuring completion of DNA duplication by the end of S-phase.

## Introduction

DNA replication is a key event in the cell cycle, occurring within a confined period termed S-phase. Replication initiates at various times at multiple sites (origins) in eukaryotic genomes [1]–[3]. Microarray analysis of enriched heavy:light nascent DNA in *Saccharomyces cerevisiae* has revealed a genomic view of DNA replication timing profiles: some regions of the genome are always replicated early in S phase, some in the middle, and others at the end, due to a strict timing of (efficient) firing at origins [4]. Similar profiles of DNA replication timing have been generated using microarrays that monitor DNA copy number increase without enrichment of nascent DNA in *S. cerevisiae* and *Drosophila melanogaster* [5], [6]. Studies in human HeLa cells have shown that more than 60% of the genome is slowly replicated throughout the S-phase, owing to a flexible timing of firing or inefficient firing at origins [7]. It has been shown that DNA replication/firing in *S. pombe* is less efficient than that in *S. cerevisiae* [8], [9]. Although inefficient origins are not readily detected by classic 2D-gel electrophoresis, a molecular combing technique that analyzes DNA replication/firing at single origins has been able to demonstrate the existence of infrequent firing events at origins in *S. pombe* [9].

In *S. pombe*, replication checkpoint controls mediated via the ATM-related kinase Rad3p (Mec1p in *S. cerevisiae*) [10]–[12] and checking DNA synthesis kinase Cds1p (Rad53p in *S. cerevisiae*) [13], [14]) play a pivotal role in restoring stalled replication forks and in the regulation of replication timing [14]–[16]. Hydroxyurea (HU) is a commonly used compound that inhibits ribonucleotide reductase (RNR) leading to a depletion of the intracellular pool of dNTPs that induces DNA replication blocks [17]. Patches (∼5 Kb in size) of single-stranded DNA (ssDNA) are found to accumulate at sites of origins upon replication blocks [18], [19]. The replication checkpoint appears to prevent the accumulation of ssDNA patches at some origins, likely the late-firing ones [20], consistent with its role in regulation of replication timing. Genome-wide profiling of enriched ssDNA induced by HU treatment has revealed several hundreds of potential origins of replication in both *S. cerevisiae* and *S. pombe* [19].

The genome-wide profiling of replication has been successfully applied to identify early-firing origins in the genome, although it has limitations in predicting the late-firing origins that are closely located to early-firing origins and especially the inefficient late-firing origins [2]. The correlation between the fold-enrichment of HU-induced ssDNA and replication efficiency has been noted for some of the origins [19] [21]. Estimation of firing efficiency by the fold-enrichment of ssDNA has been further extended to the whole genome [21], although a small number of late-firing origins have been found to exhibit a high magnitude of ssDNA formation [6], [19]. Therefore there remains a need to develop a method to directly determine the firing efficiencies of origins of DNA replication on a genomic scale.

In this study, we have used *S. pombe* genome-wide ORF-specific DNA microarrays to monitor DNA copy number increase in cells released from HU-block. A near-sigmoid model is applied for the determination of the rate of DNA copy number-increase as a function of time at individual loci across the genome. This is the first-of-its-kind study on assessing both the replication timing and efficiency at the genomic scale. We show that the rate of DNA copy number-increase in cells released after HU-arrest, is generally slow in early S-phase due to the inefficient early-firing at origins. Efficient replication appears to occur late in S-phase. Furthermore, we show that inefficient origins, but not efficient ones, are more likely to fire in *cds1Δ* cells, attributing to the remaining unused (inefficient) origins after HU removal.

## Results

### Nomenclatures

To simplify, the DNA replication process is broken down into three steps: start (*i.e.*, initiation of replication by firing and/or passive replication at individual loci), progression, and end (*i.e.*, completion of replication). We define replication start timing (*i.e.*, firing timing at origin loci or initial passive replication timing at non-origin loci), average replication timing (or half-completion timing), and (full-) completion timing as *T_0_*, *T_50_*, and *T_100_*, respectively. The time period from the start to the end is defined as the duplication time *ΔT* ( = *T_100_*-*T_0_*). As one and only one copy of DNA at all loci would be synthesized during the S-phase, *η_r_* · *ΔT* = 1, where *η_r_* is the (average) replication efficiency. The replication efficiency *η_r_* of the loci at or near the origin sites is the maximal estimate of the firing efficiency *η_f_* of the origins.

### Duplication Time Is Extended in *rad3Δ* and *cds1Δ* Cells Released after HU-block

**Figure 1:**
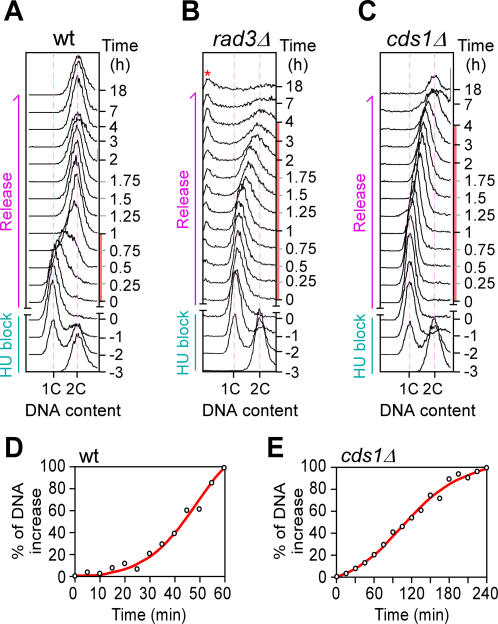
Characterization of the DNA-content increase in HU-treated cells. DNA content profiles by FACS analysis in HU-challenged wild type (A), *rad3Δ* (B), and *cds1Δ* (C) cells. 1C and 2C indicate cells containing 1 and 2 copies of genome, respectively. Minus and plus times indicate cells before and after HU-release, respectively. Regression analysis of DNA content increase in wild type (E) and *cds1Δ* (F) cells. *X*- and *Y*-axis indicate the time after HU-removal and the percent of DNA content (*i.e.*, median) increase, respectively.

Cells bearing a *rad3Δ* or *cds1Δ* allele are known to exhibit hypersensitivity to temporal treatment with HU, consistent with their replication checkpoint function. To determine whether DNA duplication could resume in *rad3Δ* or *cds1Δ* cells after temporal HU treatment, we treated log-phase growing cells for 3 hr with HU at a final concentration of 8 mM. A typical 2C-DNA content profile was observed for all strains before HU treatment, owing to a short G1-phase (Figure 1). After 3 hr of HU treatment, almost all *rad3Δ, cds1Δ*, and wild type cells showed a 1C-DNA content profile, indicating an early S-phase arrest.

DNA synthesis gradually resumed in wild type cells released after HU block, as judged by the increase in DNA content determined by FACS analysis (Figure 1A). Approximately 20% of the genome was synthesized in the first 30-min of the S-phase and the remaining 80% was completed in the second 30-min (Figure 1A and 1D), close to the time required for genome duplication in cells with undisturbed S-phase [21], [22]. The time taken for the genome duplication was ∼60 min in wild type cells released after HU-arrest (Figure 1A). In *rad3Δ* or *cds1Δ* cells, on the other hand, less than 20% of the genome was duplicated in the first 60-min after HU removal (Figure 1B, 1C, and 1E). Significantly, the time taken for the entire genome duplication was approximately 4 h in both *rad3Δ* and *cds1Δ* cells released after HU block. Given that HU-induced stalled forks collapse and fail to resume DNA replication in *rad3Δ* and *cds1Δ* cells after HU-release, this result suggests that post-HU initiation of DNA replication was likely to occur at unused origins.

It is worth noting that ∼20% of cells in the *rad3Δ* strain, but not *cds1Δ*, exhibited a cut phenotype (cells with premature assembly of division septa) at ∼2 hr after release from HU treatment (Data not shown). Consistent with this, an additional population of cells containing incomplete genomes (less than 1C-DNA contents) appeared after HU removal in the *rad3Δ* strain (Figure 1B, see asterisk). This heterogeneity of DNA content impeded determination of DNA replication profiling in *rad3Δ* cells. Therefore, genome-wide replication profiling of wild type and *cds1Δ* cells released after HU-block were performed in this study, but profiling of *rad3Δ* cells was omitted.

### Genome-wide Profiling of DNA Replication Timing and Efficiency

To investigate genome-wide DNA replication timing and efficiency, we applied the *S. pombe* genome-wide ORF-specific microarray [23] to determine the increase in DNA copy numbers at individual loci in cells released after HU block. The microarray has an average resolution of one locus/∼2.4 Kb. Each locus (or ORF) was represented by two different 50-mer oligonucleotides whose average ratio was used for profiling. Cell samples were taken at 5-min intervals after HU removal for a period of 60 min. Genomic DNA extracted from cell samples was labeled with cyanine-dye Cy5 and subjected to DNA microarray hybridization. To reduce the dye-bias in the dual-color microarray analysis, we applied a common reference of genomic DNA (Cy3-labeled) extracted from cell samples at the 0-min time point. After the 0-min normalization, profiles would not be affected by the type of the common references used (*e.g.*, G1, G2 or asynchronous cells). To ensure reproducibility, two independent time-course experiments were carried out.

Upon HU treatment for up to 3 hr, very little DNA synthesis was detected in check point-proficient cells [19]. Assuming that a replication origin could fire at ∼30% efficiency and accumulate DNA patches of ∼5 Kb in length [9], a cell containing ∼500 origins would have synthesized ∼750 Kb ( = 30%×5×500) or ∼5% of the genome during the HU treatment. The vast majority (∼95%) of the remaining genome would be synthesized in cells released after HU block. Therefore, the tiny amount of HU-induced DNA deposits at origins is unlikely to distort the global profiles of replication timing and efficiency after the smoothing process (see Materials and Methods).

**Figure 2:**
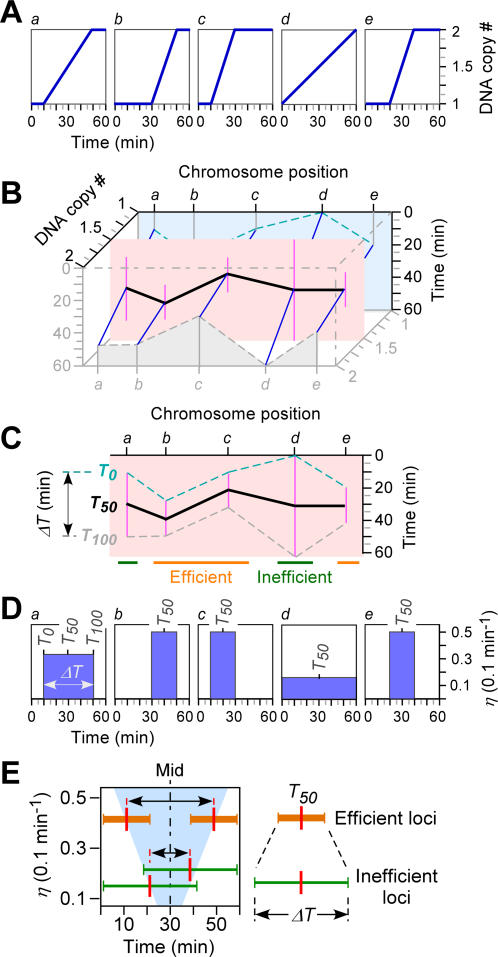
Schematic modeling of the near-sigmoid regression for profiling of DNA replication timing and efficiency. (A) Modeling of the near-sigmoid regression for the increase in DNA copy number at indicated individual loci (a–e). (B) Profile assembly of the copy number changes at individual loci of the subchromosomal region. (C) Simplified form of replication profiles displaying average replication timing *T_50_* and duplication time *ΔT*. (D) Conversion between duplication time *ΔT* and replication efficiency *η*. (E) Potential distribution of the *T_50_* versus *η* at individual loci of the genome.

**Figure 3:**
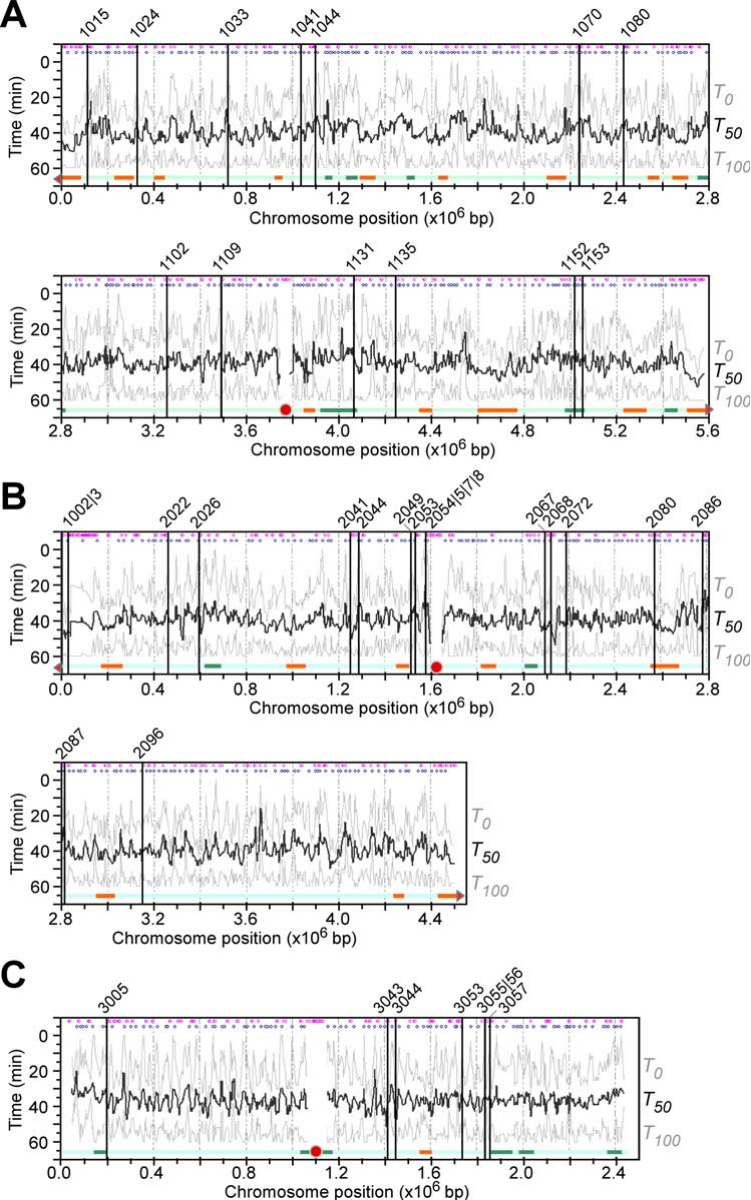
Global profiles of DNA replication timing and efficiency in wild type cells released from HU block. X- and Y-axis are times after HU removal and physical locations of individual loci of chromosome *I* (A), *II* (B), and *III* (C), respectively. Average replication timing *T_50_* profiles are in think black lines. Efficiency is indicated by *T_0_* and *T_100_* profiles at individual loci in grey lines. Approximately 39 origins that have been validated by 2D-gel electrophoresis are marked with vertical lines. Predicted origins and AT-rich islands are indicated in blue and purple dots on the top of the profiles, respectively. Horizontal orange and green lines at the bottom of the profiles indicate efficient and inefficient subchromosomal regions, respectively. Solid red circles and triangles indicate centromeres and telomeres. No adjacent telomeres indicated in chromosome *III* due to the presence of multiple arrays of large rDNA sequences at the ends.

We applied the near-sigmoid model to fit DNA copy number increase (after 0-min normalization) as a function of time at individual loci for estimation of replication start timing *T_0_* and end timings *T_100_* (exemplified in Figure 2). To this end, the *T_0_* and *T_100_* at the vast majority of loci (>96%) were obtained based on the threshold of FDR (false discovery rate) less than 0.01%. Approximately 190 loci (∼4%) of the genome could not be fit to the near-sigmoid model (see Materials and Methods). After smoothing with a moving window of 3 loci, genome-wide profiles of the average replication timing *T_50_* and the duplication time *ΔT* (or replication efficiency *η_r_*) were revealed (Figure 2 and 3; Table S1).

We defined loci whose replication efficiency *η_r_* was greater than the median *η_r_*_-*median*_ (or duplication time *ΔT* was less than the median *ΔT_median_*) as efficient-replication loci or efficient loci (Figure 2C, loci *b, c,* and *e*). On the other hand, loci whose *η_r_* was less than *η_r-median_* were known as inefficient loci (Figure 2C, loci *a* and *d*). It is worth noting that inefficient loci would need to start replication early (through firing at origins and/or passive replication) in order to complete DNA duplication prior to the end of S-phase. This, the *T_50_* at inefficient loci should have a limited range around half the length of the S-phase (Figure 2D and 2E). On the other hand, the *T_50_* at efficient loci would have wide range from early to late in S-phase (Figure 2E). A volcano-like shape illustrates the potential distribution of average-replication timings for all loci with various replication efficiencies (Figure 2E).

### Average Replication Timing at Efficient Loci Appears to Be Late in S-phase Progression in Cells Released after HU-block

**Figure 4:**
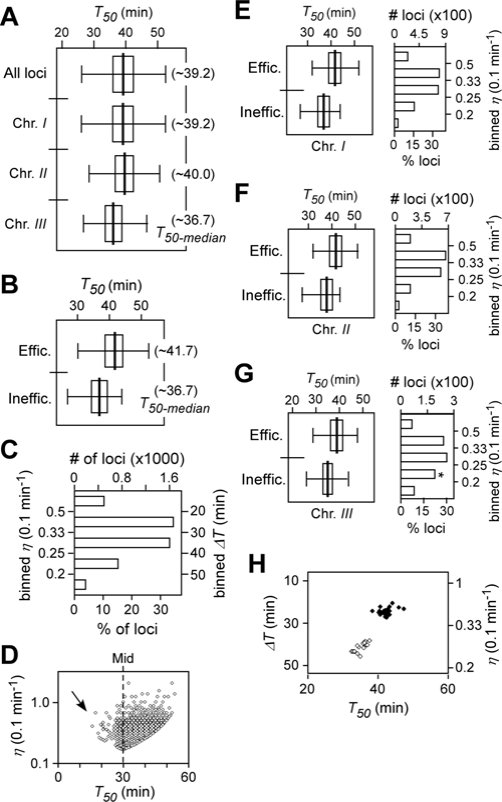
Characteristics of DNA replication timing *T_50_* and efficiency *η* at various loci in wild type cells released after HU block. Box plot indicates the non-outliers minimum (left end of the line), the lower quartile (left end of the rectangle), median (centre of the rectangle), the upper quartile (right end of the rectangle), and the non-outlier maximum (right end of the line). (A) The box-plots of *T_50_* distribution of individual loci of the genome and individual chromosomes as indicated. The *T_50-median_* of the loci of the genome or individual chromosomes is presented in parentheses. (B) The box-plots of *T_50_* distribution of efficient and inefficient loci. Efficient and inefficient loci are those whose *η* is greater and less than the *η_-median_*, respectively. The *T_50-median_* of efficient and inefficient loci is indicated in parentheses. (C) Numbers of loci in the binned *ΔT* series or *η* series. (D) Plot of efficiency *η* versus average replication timing *T_50_* of individual loci. Only ∼0.3% (16 out of 4733) loci of the genome exhibited their efficiency *η* of greater than 1 (or duplicating the DNA in less than 10 min). The arrow indicates the asymmetric distribution of the *T_50_*. (E–G) The *T_50_* distribution of loci and the number of loci in the binned *η* series in chromosome *I* (E), *II* (F), and *III* (G). It is displayed as described in (B) and (C). (H) The plot of *T_50_* versus *η* or *ΔT* of efficient and inefficient regions. *X*- and *Y*-axis indicate *T_50_* and *η* or *ΔT*, respectively. The efficient and inefficient regions are indicated by closed and open circles, respectively.

The profiles of average replication timing *T_50_* and efficiency *η* (*ΔT* was also included in Figure 3) revealed that the *T_50_* at all loci of the genome ranged from ∼25.8 to ∼52.5 min with a median of ∼39.2 min at various efficiencies (Figure 4A). The average replication timing *T_50_* varied slightly in different chromosomes. The median of average replication timing *T_50-median_* was ∼39.2 and ∼40 min in chromosome *I* and *II*, respectively. On the other hand, the *T_50-median_* in chromosome *III* was ∼36.7 min, >2.5 min earlier than the other two chromosomes (*p*-value ∼ 2.2×10^−16^) (Figure 4A). This result is consistent with the observation by Heichinger et al. [21].

We next investigated replication efficiency *η_r_* at various loci of the genome. We set the unit of the replication efficiency *η_r_* as 0.1 min^−1^. Loci whose *η_r_* was greater that the median *η_r-median_* of ∼0.316 (0.1-min^−1^) were designated as efficient-replication loci or efficient loci. Conversely, inefficient loci were those whose *η_r_* was less than *η_r-median_*. Surprisingly, the median *T_50-median_* of the efficient loci (*i.e.*, ∼41.7 min) was substantially later (or greater) than the *T_50-median_* of the inefficient loci (*i.e.*, ∼36.7 min), indicating that efficient replication tends to occur late in S-phase (Figure 4B–D; Table 1). The late efficient-replication was a common feature in all three chromosomes (Figure 4E–G). It is probably not surprising that cells would need to synthesize DNA more efficiently in unreplicated loci or gaps late in S-phase. Otherwise a delay in S-phase would be inevitable.

**Table 1:** The medians of replication/firing timing and efficiency at loci/origins

Strain	Loci	No.	*ΔT_-median_*	*η_-median_^a^*	*T_50-median_*
**wt**	All loci	4733	39.2 (min)	0.255 (0.1 min^−1^)	31.7 (min)
	Pk. loci^b^	516	31.6 (min)	0.316 (0.1 min^−1^)	36.5 (min)
***cds1***	All loci	4787	150 (min)	0.267 (0.025 min^−1^)	100 (min)
	Pk. loci	598	145 (min)	0.276 (0.025 min^−1^)	95 (min)

Note: ^a^It stands for the median of either replication efficiency *η_r-median_* or firing efficiency *η_f-median_* where appropriate; Replication/firing efficiency is defined as 1/10 min^−1^ in wild type and 1/40 min^−1^ in *cds1Δ*. ^b^Pk or peak loci indicate the loci at or near the predicted peaks/origins of replication.

To investigate regulation of replication in subchromosomal regions, we searched for regions (*e.g.*, ∼20 consecutive loci or more) that were enriched either efficient or inefficient loci (*p*-value<0.05) in the genome. To this end, 23 and 14 subchromosomal regions were found to be efficiently and inefficiently replicated, respectively (Figure 3, see orange and green bars). Inefficient replication regions (5 out of 14) were found to be over-represented in chromosome *III* (*p*-value<0.02) (Figure 3C), consistent with the observation that DNA replication is less efficient on chromosome *III* compared to the other two chromosomes. On the other hand, subtelomeric regions (3 out of 4) in chromosome *I* and *II* (chromosome *III* telomeres are separated from the arms by the multiple arrays of rDNA) were identified as efficient regions that replicated late (Figure 3), consistent with the report on late replication in subtelomeric regions [8]. The average replication timings *T_50_* of the efficient-replication regions appeared to be remarkably later than the *T_50_* of the inefficient regions (*p*-value<10^−15^) (Figure 4H).

### Chromosomal Locations, Firing Timings and Efficiencies of Potential Replication Origins

Peaks identified from the profiles of average replication timing *T_50_* were commonly used to predict the location of replication origins (or origin clusters). Timing of firing at origins is approximated by the average replication timing of the peak loci. It has been noted that this approach has limitations in identifying (efficient) late-firing origins closely located to the other (efficient) early-firing origins [2], [4]. As firing at origins is relatively inefficient in *S. pombe* [8], [9], [24], it would not only underestimate the number of efficient late-firing origins, but also would fail to identify most, if not all, inefficient late-firing origins. This is because inefficient late-firing origins are unlikely to be self-sufficient in replication of the origin DNA (see Material and Methods). Nevertheless, this is still the most effective way to predict origins of replication at the genomic scale [21].

The PeakFinder software [25] was applied to identify the position of peaks or potential origins based on the profiles of average replication timing. To this end, ∼516 peaks were identified that represent potential origins (or origin clusters) of replication in the genome (Figure 3, see blue dots on the top of the replication profiles). Of the 48 origins that have been tested by 2D-gel electrophoresis (see ref in Segurado), 39 (∼81%) were found to overlap with the peaks identified in this study with a window of 12-Kb distance (Table 2), and the remaining 15 origins were found to match with peaks a bit below the threshold, suggesting that the peaks of the *T_50_* profiles derived from the *T_0_* and *T_100_* are good approximations of potential origins in the genome.

**Table 2:** The comparisons of the peaks identified in *T_50_* profiles with the ORI sites reported previously by others

Type of ORI studies	# Total^a^	# OL^b^	% OL	Reference
2D-gel tested ORI sites	47	43	91%	Ref. in Segurado *et al.*, (2003)
A+T-rich islands	365	324	89%	Segurado *et al.*, (2003)
Orc1-binding sites	447	386	86%	Hayashi *et al.*, (2007)
Enriched ssDNA sites	241 (321)^c^	224 (299)	92% (93%)	Feng *et al.*, (2006)
ORI in undist. S-phase	401	378	94%	Heichinger *et al.*, (2007)

Note: ^a^Numbers of ORI are referred to those in the genome except telomeric or centromeric regions. ^b^OL stands for overlapping origins with a window of 12-Kb distance. ^c^Numbers in parenthesis are those predicted in *cds1Δ* cells.

**Figure 5:**
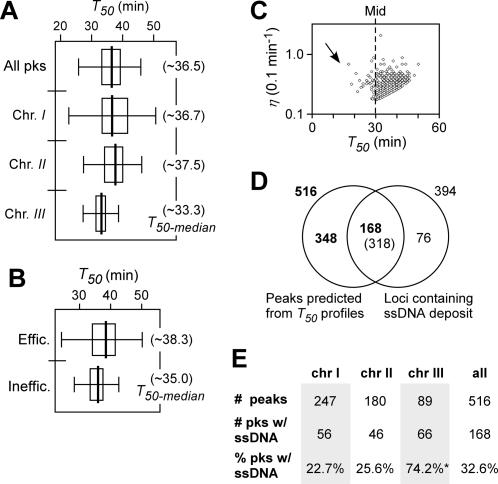
Characteristics of average firing timing *T_50_* and (maximum) firing efficiency *η* at various predicted origins in wild type cells released after HU block. (A) The box-plots of *T_50_* distribution of predicted origins of the genome and individual chromosomes as indicated. The *T_50-median_* of the origins of the genome or individual chromosomes is presented in parentheses. (B) The box-plots of *T_50_* distribution of efficient and inefficient origins. Efficient and inefficient origins are those whose *η* is greater and less than the *η_-median_*, respectively. The *T_50-median_* of efficient and inefficient origins is indicated in parentheses. (C) Plot of firing efficiency *η* versus average firing timing *T_50_* of individual origins. The arrow indicates the asymmetric distribution of the *T_50_*. (D) The Venn diagram of predicted peaks and loci containing HU-induced ssDNA deposit. (E) The over-representation of ssDNA in predicated origins of chromosome *III*. Numbers of the predicated origins/peaks and those overlapped with the loci containing ssDNA deposit are indicated. The asterisk indicates the significant enrichment of ssDNA in chromosome *III*.

We estimated the average firing timings *T_50_* and the (maximum) firing efficiencies *η_f_* at the predicted origins using the average replication timing *T_50_* and replication efficiency *η_r_* of the peak loci (Figure 5A). Origins whose firing efficiency *η_f_* was greater than the median *η_f-median_* of 0.316 (0.1 min^−1^) were designated as efficient origins (the *η_f-median_* is different from the *η_r-median_*) (Table 1). On the other hand, origins whose *η_f_* was less than the *η_f-median_* were classified as inefficient origins. Notably, most inefficient origins fired near the middle of the S-phase (Figure 5B and 5C). It is intriguing that most efficient origins appeared to fire late in S-phase in cells released after HU block (Figure 5B and 5C), given that the method applied was known to favor early-firing origins [2],

Rad3p-Cds1p mediated checkpoint is known to restore stalled forks during HU-induced DNA replication block [9], [15], [19]. To validate that the majority of the stalled forks resumed DNA replication in cells released from a HU-block, we first determined the sites of DNA synthesis in cells treated with 8-mM HU for 3 h (equivalent to the cells at 0-min time point). To achieve this, microarray analysis was performed using Cy5-labeled sample DNA prepared from HU-treated cells (*i.e.*, 0-min samples) against Cy3-labeled reference DNA from G1- or G2-phase cells. Self-hybridizations of genomic DNA from G1 or G2-phase cells were used as control. In either case, four independent microarray hybridizations were carried out for reproducibility. Loci containing extensive stretches of newly synthesized DNA, resulting from firing at origins in HU-treated cells, would exhibit an obvious increase in DNA copy number. SAM procedure [26] was applied to identify loci with significant amounts of nascent DNA by comparing the ratios of copy number at individual loci in HU-treated experiments with the self-hybridization control experiments. As a result, ∼394 loci were found to have large amounts of newly synthesized DNA at the threshold of 10% FDR (Figure 5D). More than 80% of the loci containing nascent DNA after HU-release overlapped with the predicted origins derived from the peaks of the *T_50_* profiles with a window of 12-Kb distance, indicating that the majority of the stalled forks are rendered competent for DNA replication in checkpoint-proficient cells released from a HU block. The HU-induced nascent ssDNA stretches were found to be over-represented on chromosome *III*, consistent with the observation of early replication/firing in chromosome *III* (Figure 5E, see asterisk).

To investigate whether the peaks derived from the *T_50_* (*i.e.*, the average of *T_0_* and *T_100_*) profiles were similar to the origins predicted by other studies, we compared the 516 peaks with the reported origin sets based on an *in-silico* study [27], a chIP-chip analysis [28], an HU-induced ssDNA enrichment [19], and from replication profiling in unperturbed S-phase [21] (Table S2). The majority (∼80%) of the origins in the previous reports overlapped with the 516 peaks, indicating an adequate prediction of origins using the near-sigmoid approach (Table 2).

### Late Efficient Replication Patterns Are Disrupted in HU-challenged *cds1Δ* Cells

**Figure 6:**
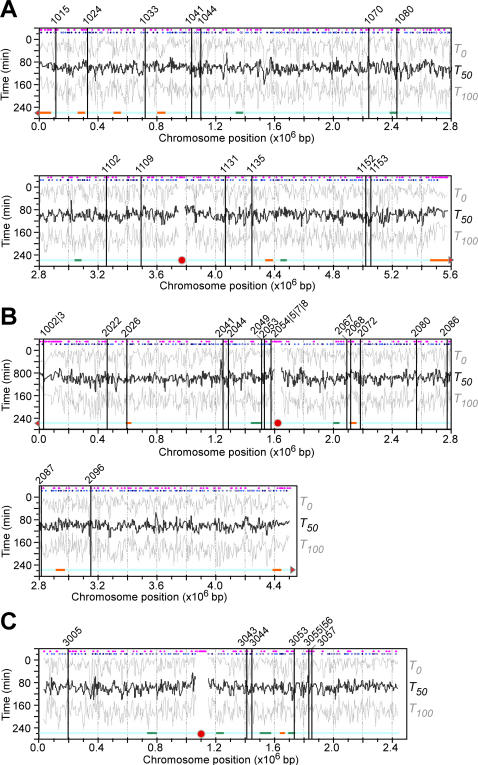
Global DNA replication profiles of HU-challenged *cds1Δ* cells. The profiles are displayed as described in Figure 3.

To investigate the role of Cds1p-mediated checkpoint function in regulation of global DNA replication, we performed DNA replication profiling in *cds1Δ* cells released from a HU block. To this end, HU-challenged *cds1Δ* cells were sampled at 15-min intervals for a period of 4 h. Microarray analysis of DNA copy number-increases in *cds1Δ* cells was performed using an identical method for the wild type (see above). Genome-wide profiles of DNA replication timing and efficiency in *cds1Δ* cells are shown in Figure 6 (Table S3). It took ∼240 min to complete DNA replication in *cds1Δ* cells released from a HU block, 4 times longer than HU-challenged wild type cells. The profiles in *cds1Δ* cells were disrupted from those in wild type cells, indicating that Cds1p function is involved in maintaining global regulation of DNA replication.

**Figure 7:**
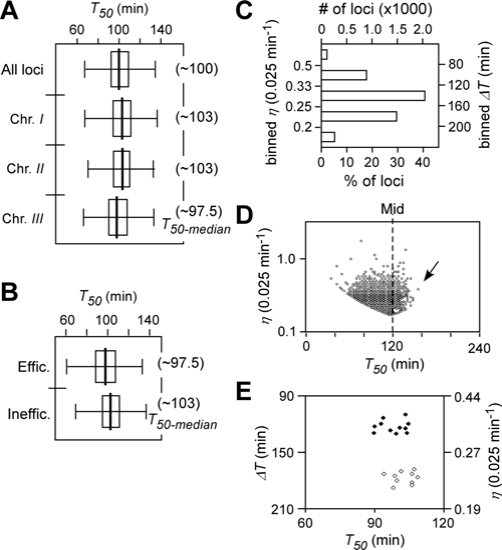
Characteristics of DNA replication timing *T_50_* and efficiency *η* at various loci in *cds1Δ* cells released after HU block. (A) The box-plots of *T_50_* distribution of individual loci of the genome and individual chromosomes in *cds1Δ* cells as indicated. The *T_50-median_* of the loci of the genome or individual chromosomes is presented in parentheses. (B) The box-plots of *T_50_* distribution of efficient and inefficient loci in *cds1Δ* cells. (C) Numbers of loci in the binned *ΔT* series or *η* series. (D) Plot of efficiency *η* versus average replication timing *T_50_* of individual loci in *cds1Δ* cells. Less than ∼0.1% (3 out of 4787) loci of the genome exhibited their efficiency *η* of greater than 1 (or duplicating the DNA in less than 40 min). The arrow indicates the asymmetric distribution of the *T_50_*. (E) The plot of *T_50_* versus *η* or *ΔT* of efficient and inefficient regions in *cds1Δ* cells. *X*- and *Y*-axis indicate *T_50_* and *η* or *ΔT*, respectively. The efficient and inefficient regions are indicated by closed and open circles, respectively.

It was clear that the average replication timing *T_50_* at various loci ranged from ∼70 min to ∼135 min with the median *T_50-median_* of 100 min according to the replication profiles in HU-challenged *cds1Δ* cells (Figure 7A). The global profiles were totally disrupted in *cds1Δ* cells. However, the early replication of chromosome *III* (*i.e.*, *T_50-median_* = ∼97.5 min) compared to that of the other two chromosomes was not altered in *cds1Δ* cells (Figure 7A), suggesting that the early replication of chromosome *III* is independent on the Cds1p function. As DNA duplication in HU-challenged *cds1Δ* cells took ∼4 times longer than that of wild type cells, the unit of replication efficiency *η_r_* was thus defined as 1/40 (or 0.025) min^−1^, four-fold less efficient than wild type cells. The relative replication efficiency at various loci in *cds1Δ* cells was categorized based on the median *η_r-median_* ∼0.267 (0.025 min^−1^). The *T_50-median_* of efficient loci in *cds1Δ* cells was very similar to that of inefficient loci (Figure 7B–D), indicating the late efficient-replication origins were most affected by the lack of Cds1p after release from a HU block (Figure 4B–D). This result suggests that the failure in resuming DNA replication at the forks that previously initiated DNA replication, would extend the duration of S-phase. Alternatively, it may imply that the checkpoint function is not only required for restoring stalled forks, but also involved in ensuring efficient replication late in S-phase.

We subsequently searched for subchromosomal regions that were clustered for efficient and inefficient replication loci in *cds1Δ* cells using the identical approach in wild type. We identified 11 efficient and 10 inefficient regions. Inefficient replication regions were over-represented on chromosome *III*, similar to wild type cells. The average replication timing, *T_50,_* of efficient regions was comparable to that of inefficient regions in *cds1Δ* cells (Figure 7E). The late-efficient replication pattern in wild type was clearly disrupted in *cds1Δ* cells, indicating that the involvement of Cds1p function in maintaining the efficient replication late in S-phase.

### Checkpoint Function Is More Apparent on Efficient Origins than Inefficient Ones in *cds1Δ* cells Released From a HU-block

**Figure 8:**
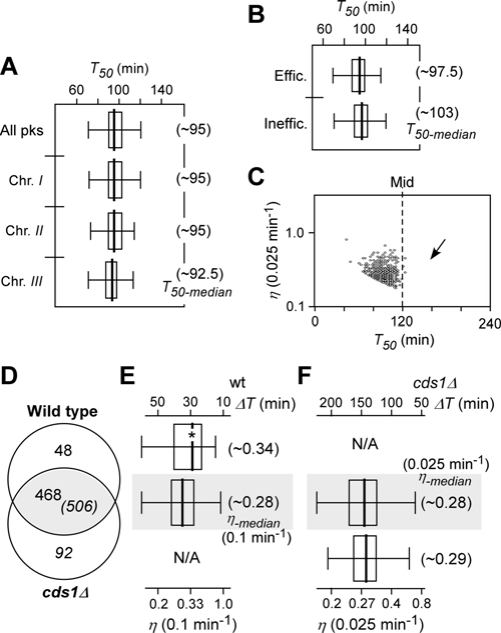
Characteristics of average firing timing *T_50_* and (maximum) firing efficiency *η* at various predicted origins in *cds1Δ* cells released after HU block. (A) The box-plots of *T_50_* distribution of predicted origins of the genome and individual chromosomes *cds1Δ* cells as indicated. The *T_50-median_* of the origins of the genome or individual chromosomes is presented in parentheses. (B) The box-plots of *T_50_* distribution of efficient and inefficient origins *cds1Δ* cells. The *T_50-median_* of efficient and inefficient origins is indicated in parentheses. (C) Plot of firing efficiency *η* versus average firing timing *T_50_* of individual origins *cds1Δ* cells. The arrow indicates the asymmetric distribution of the *T_50_*. (D) Venn diagram of peaks predicted in wild type and *cds1Δ* cells. Overlapping peaks are those whose distance is less than 12 Kb. (E) The *T_50_* distribution of the wild type-unique and overlapping origins in wild type cells. The asterisk indicates the significant increase in firing efficiency of the wild type-unique origins. (F) The *T_50_* distribution of the cds1-unique and overlapping origins in *cds1Δ* cells.

Chromosomal locations of origins were approximated by the peaks identified using the Peakfinder software [25] based on the profiles of the average replication timing *T_50_* in *cds1Δ* cells. A total of 598 peaks were identified from the profiles in HU-challenged *cds1Δ* cells: 269, 226, and 103 were found in chromosome *I, II*, and *III*, respectively (Figure 8A–8C). The number of origins identified in *cds1Δ* profiles was ∼14% more than that found in wild type profiles.

It has been shown that Cds1p-mediated checkpoint is required for restoring stalled forks in cells treated with HU [15]. Stalled forks would collapse in *cds1Δ* cells and fail to resume replication. Assuming that the same subset of origins has fired and gave rise to nascent ssDNA stretches of ∼5 Kb in size in either wild type or *cds1Δ* cells during HU treatment, we would expect that the stalled forks close to the origins would be able to resume replication in wild type cells but not in *cds1Δ* cells, representing a subset of the wild type-unique origins.

To identify the wild type-unique origins, chromosomal location of origins in wild type cells was compared to that in *cds1Δ* cells using the threshold of 12 Kb-distance (see Materials and Methods). Surprisingly, less than 10% origins (48 out of 516) were found to be wild type-unique (Figure 8D). The majority of the predicted origins were found to overlap between the wild type and *cds1Δ* cells with the window of 12-Kb distance (Figure 8D). It is very unlikely that the majority of origins would have been reserved (not fired) during HU treatment (for 3 h) and could fire in either strain after HU removal. Given that firing efficiency at origins in general is low in *S. pombe* [9], it is possible that those overlapping origins represent a subset of inefficient origins.

To test whether overlapping origins were inefficient, we performed comparison analysis of the firing efficiencies between the two origin-subsets: the wild type-unique and the overlapping origins. As expected, the firing efficiency of the wild type-unique origins appeared to be higher than that of overlapping ones (p-value ∼7.3×10^−3^), consistent with the notion that Cds1p checkpoint function is essential for restoring stalled forks induced by HU treatment (Figure 8E). The result indicates that checkpoint function has a greater effect on efficient origins compared to inefficient ones. A small subset of *cds1*-unique origins was found in *cds1Δ* cells (Figure 8F). These *cds1*-unique origins might represent the late inefficient origins which failed to be identified in wild type cells.

## Discussion

Genome-wide microarray analyses have been widely used to determine profiles of average DNA replication timing at the genomic scale [4]–[7], [21]. However, direct estimation of replication efficiency at various loci of the genome based on the genome-wide replication profiles has not been performed previously. In this study, we demonstrate that replication efficiency, together with average replication timing, can be estimated using a novel approach-the near-sigmoid fitting for the increase in DNA copy number as a function of time at individual loci (see Materials and Methods). The near-sigmoid model approach permits estimation of replication start timing *T_0_* and replication end timing *T_100_* at various loci of the genome. Based on the *T_0_* and *T_100_* at each locus, we attain the genome-wide profiles of average replication time *T_50_* (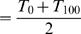) and replication efficiency *η* (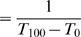).

Genome-wide profiles of average replication timing *T_50_* and replication efficiency *η* were determined in wild type and *cds1Δ* cells released from a HU block (Figure 3 and Figure 6). DNA duplication takes about ∼60 min in wild type cell and ∼240 min *cds1Δ* cells after HU removal (Figure 1). The time taken to complete duplication at the majority of loci ranges from ∼15.8 min to ∼53.8 min with the median of 39.2 min in wild type cells and from ∼37.5 min to ∼157.5 min with the median of 150 min in *cds1Δ* cells. The relative rate of DNA copy number-increase is used to define replication efficiencies in wild type (*i.e.*, 1/10-min^−1^) and *cds1Δ* (*i.e.*, 1/40-min^−1^) cells. The medians of replication efficiency *η_r-median_* of 0.255/10-min and *η_r-median_* of 0.267/40-min are used to categorize efficient or inefficient loci in wild type and *cds1Δ* cells, respectively (Table 1). Significantly, efficient loci (whose *η*>0.255/10-min) in wild type cells tend to be replicated late while inefficient ones (whose *η*<0.255/10-min) early in S-phase (Figure 4). However, efficient loci (*η*>0.267/40-min) in *cds1Δ* cells do not show late replication when compared to inefficient loci (*η*<0.267/40-min) (Figure 7). It is not clear whether the disruption of the late-efficient replication in *cds1Δ* cells is solely due to the failure in resuming replication from the collapsed forks. Alternatively, Cds1p checkpoint kinase may play a role in regulation of late-efficient replication in cells after HU removal.

It is intriguing to find that efficient replication occurs late in S-phase. To ensure completion of DNA duplication by the end of S-phase in an inefficient replication system such as *S. pombe* [9], [29], it is necessary to increase the efficiency of origins that fire late in S-phase. Disruption of the late efficient replication would lead to a delay in completion of duplication or S-phase. It is most likely that the extended S-phase in *cds1Δ* cells after HU removal is partly attributed to the disruption of the late efficient replication.

To assess whether the HU-induced newly-replicated DNA would distort the profiles of replication timing and efficiency in cells released from a HU block, we performed microarray analysis to compare DNA extracted from HU-treated cells and G1 or G2 cells. Assuming that ∼500 origins fire at an average efficiency of ∼30% to produce newly-replicated DNA stretches of ∼5 Kb in size [9], ∼5% of the genome would have been synthesized after HU treatment for 3 h. Approximately 394 loci are found to have newly-replicated DNA, out of which, 318 (80%) co-localize to the peaks of the *T_50_* profiles with a window of 12-Kb distance. This indicates that the replication/resumption profiles generated based on the common reference of 0-min samples are a fair representation of the actual replication profiles.

Peaks identified from the global profiles of DNA replication timing *T_50_* have been commonly used to estimate origins of replication [2], [4]. We have adopted this approach to identify peaks from the *T_50_* profiles in wild type and *cds1Δ* cells. A total of 516 and 598 peaks were identified in wild type and *cds1Δ* cells, respectively. Origins that initiated DNA replication prior to HU treatment could resume replication in wild type cells after HU removal. These origins would presumably be readily identifiable. However, forks resulting form origins that initiated DNA replication prior to HU treatment would fail to resume replication in *cds1Δ* cells due to collapse and therefore are unlikely to be identified. To investigate whether we identified different sets of origins in wild type and *cds1Δ* cells, we compared the actual locations of peaks in wild type and *cds1Δ* cells. Significantly, the majority of peaks overlapped with a window of 12-Kb distance between the two sets of origins (Figure 8). This may not be surprising in *S. pombe* in which firing is inefficient; and initiation of DNA replication from inefficient origins would not be effective during HU treatment. Therefore, after HU removal, the remaining unused origins could be competent to initiate DNA replication in both wild type and *cds1Δ* cells. This is further supported by the observation that origins found in wild type but not in *cds1Δ* cells are efficient origins (Figure 8E). These results are consistent with the notion that replication in *S. pombe* is generally considered inefficient [9].

Heichinger *et al.*, has recently reported the profiling of average DNA replication timings using *cdc25-22* cells after temperature block-and-release [21]. The firing efficiency at various origins is estimated based on the fold-enrichment of HU-induced ssDNA. It was thus concluded that the early-firing origins are more efficient that the late-firing ones. The discrepancy may be explained by the fact that the late-firing origins are inefficient in cells undergoing unperturbed S-phase. However, in checkpoint activated cells, DNA replication from efficient origins are delayed.

We present a first-of-its-kind study that permit direct assessment of genome-wide replication/firing efficiency at individual loci/origins. Opposed to well-defined, site-specific, and efficient (∼90%) origins in budding yeast, origins of replication in *S. pombe* appear to be located preferentially at A+T-rich regions in the genome with an average firing efficiency of ∼30% [9], [27], [29]. Approximately 50% of the loci have shown to possess a low level (below the threshold) of HU-induced newly replicated DNA (Eshaghi and Liu, unpublished data), supporting the idea that about half of the genome (*i.e.*, all intergenic sequences) have regions that could potentially act as sites for initiation of DNA replication [29]. Our results support the stochastic model [24], [30] in which random gaps are likely to be filled by the efficient late-firing origins (Figure 5).

## Materials and Methods

### Strains and Culture Manipulations

Strains YJL188 (*leu1-32 ura4-D18 h^−^*), YJL1687 (*leu1-32 ura4-D18 cds1Δ::ura4^+^ h^−^*), YJL1715 (*leu1-32 ura4-D18 rad3Δ::ura4^+^ h^−^*), and medium YES [31] were used in this study.

To monitor DNA replication in hydroxyurea (HU) (Sigma-Aldrich Corp. St. Louis, MO)-challenged cells, ∼800 ml of *S. pombe* culture in YES supplemented with 8mM HU were grown at 30°C in an orbital rotating shaker (New Brunswick Scientific Co., Inc., Edison, NJ) for 3 hrs. The HU-challenged cells were harvested by centrifugation (Thermo Scientific, Inc., Waltham, MA) at 5,000 rpm, 4°C for 1 min and subsequently washed twice with 4°C pre-chilled YES. The cells were resuspended to the original volume of YES medium. Samples were taken at 5-min intervals for a period of 60 min. For DNA content analysis, cell samples (∼5 ml each) were spun down, resuspended in ice-cold 70% ethanol and stored at 4°C for subsequent analysis. For genomic DNA extraction and analysis of copy number, cell samples (∼40 ml each) were spun down, quickly chilled in liquid nitrogen and stored at −80°C for further analysis.

### Fluorescence-associated Cell Sorting (FACS) Analysis

To analyze DNA content, cells were fixed in ice-cold 70% ethanol spun down and resuspended in 50 mM sodium citrate buffer pH 7.0. To remove RNA, the cell suspension was treated with RNase A (Sigma) at a final concentration of 100 µg/ml and left at room temperature overnight. Cells were subsequently washed with 50 mM sodium citrate buffer and stained with propidium iodine (Sigma) at a final concentration of 8 µg/ml. Fluorescence intensities of individual cells were measured by BD FACScan flow cytometry (BD Biosciences, Franklin Lakes, NJ).

### Genomic DNA preparation

The frozen cell samples were thawed and washed with SE buffer (1.2 M sorbitol/0.1 M EDTA pH 8). The cells were subsequently resuspended in 1 ml SE buffer supplemented with 2 µg/ml Zymolyase 100T (ICN Biomedicals, Inc., Costa Mesa, CA) and incubated at 37°C for ∼15 min. The spheroplasts were centrifuged at 2,000 rpm for 5 min and the supernatant was decanted. The spheroplasts were resuspended in 1 ml TNE buffer (50 mM Tris-HCl/100 mM NaCl/50 mM EDTA pH8) supplemented with 0.5% SDS and 1 µg/ml protease K (Roche, Basel, Switzerland) and incubated at 65°C for 30 min. To precipitate proteins, the cell lysates were first chilled on ice, mixed with pre-cooled 100 µl of 5 M Potassium Acetate, and incubated on ice for 30 min. Proteins were removed by centrifuging at 14,000 rpm for 15 min. The supernatant containing DNA was transferred into fresh tubes. The DNA was precipitated by the addition of equal volume of isopropanol and incubated at room temperature for 30 min. DNA was centrifuged and washed with 70% cold ethanol. The DNA pellet was subsequently resuspended in TE buffer supplemented with 0.1 µg/ml RNase A (Roche) and incubated at room temperature for 1 hr. DNA was extracted by phenol/chloroform/isoamyl alcohol (25∶14∶1) (Fluka Chemical Corp, Milwaukee, WI) extraction and precipitated by ethanol or isopropanol. The precipitated DNA was resuspended in TE buffer and fragmented by sonication (Branson, Danbury, CT) at 20% strength using three 20 sec bursts.

### Microarray Manufacture and Hybridization

To manufacture ORF-specific DNA microarrays, approximately 10,000 oligonucleotides (50-mer in length) that represented 4,929 ORFs (one ORF is represented by two oligomers) in the *S. pombe* genome [32] were synthesized (Proligo, Hamburg, Germany). The oligonucleotides were resuspended in 0.3× SSC buffer and spotted onto poly-lysine-coated glass slides by using an arrayer machine (GeneMachines, San Carlos, CA). Spotted glass slides were processed according to DeRisi's protocol (http://derisilab.ucsf.edu).

Sheared DNA was labeled using a random-priming protocol with amino-allyl-dUTP (aa-dUTP) using the BioPrime DNA Labeling kit (Invitrogen Corporation, Carlsbad, CA) according the manufacture's instruction. Labeled DNA was extracted with phenol/chloroform/isoamyl alcohol and precipitated with ethanol or isopropanol. The labeled DNA was then coupled with cyanine dyes Cy5 (Amersham, Buckinghamshire, UK) (e.g. DNA samples at various time points after HU removal) or Cy3 (e.g. DNA samples at the 0-min time point as common reference) according to a standard protocol. Cy5 and Cy3 labeled DNA were co-hybridized to microarrays.

### Microarray Data Acquisition and LOWESS Normalization

Microarray slides were scanned using a GenePix scanner (Axon Instruments, Union City, CA) that was controlled by the software GenePix Pro4 (Axon Instruments). GenePix Results files were generated and globally normalized based on a median of ratios. To ensure accuracy of the measured data, the ratio of a feature was collected if its intensity in either channel was two-fold or greater than that of the background. To eliminate intensity-dependent dye bias, individual microarrays were further normalized using locally weighted linear regression and smooth scatter plot (LOWESS) [26].

### Genome-wide Profiling of DNA Replication Timing and Efficiency

All steps in the process (including DNA-content-based microarray data normalization, regression of near-sigmoidal models to estimate replication initiation timing, completion timing and prediction of peaks using PeakFinder) were performed as follows.

#### DNA-content-based data normalization

To determine DNA copy number increase at various loci, individual microarrays were normalized based on the DNA content at respective time points as described elsewhere [6]. In brief, the DNA content profiles were obtained through FACS analysis in two independent time series experiments. Each series was first normalized to 0-mean unit-variance to eliminate series dependent variation of mean and variance. Subsequently the logistic regression was carried out using SigmaPlot 8.0 software (Systat Software Inc., San Jose, CA) in which we fitted a 4-parameter near-sigmoid curve shown (Figure 1D and 1E) by equation (1) for the two normalized DNA content series together by estimating the parameters using least squares method.
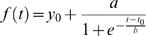
(1)Where *f(t)* is the estimated DNA-content at time “*t*”. *a* is the scale parameter; *b* is the rate of DNA content increase; *t*_0_ is the replication half-completion timing; and *y*_0_ is the initial DNA content. Considering that the initial DNA content and the amount of increase of DNA after replication completion is from 1 copy to 2 copies of genome, we therefore simplified the equation (1) as
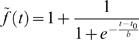
(2)Where *y*_0_ = 1 and *a* = 1 in (1). The median of ratios of individual microarrays was adjusted to be the logarithm of the estimated DNA content at respective time points from equation (2). That is,
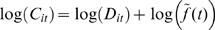
(3)Where *D_it_* and *C_it_* are ratios of locus *i* at time *t* before and after the normalization by equation (3), respectively. Normalized datasets were applied for further analysis of replication timing and efficiency.

#### Regression analysis using the near-sigmoidal model

To estimate replication initiation and completion timings, the increase of copy number at individual loci, as a function of time, was subjected to a regression fitting for a 3-piece linear or near-sigmoidal model as an approximation of the sigmoidal. Before regression analysis, the datasets was first 0-mean and unit-variance normalized. Subsequently, missing values were filled by averaging neighbor loci log-ratios for regression analysis. In brief, each locus' log-ratio was fitted to a near-sigmoidal model as in equation (4) using the least square method. *T_0_* and *T_100_* indicate replication initiation timing and completion timing, respectively. Thus, *T_50_* is the average of *T_0_* and *T_100_*, *i.e.*, (*T_0_*+*T_100_*)/2. The duplication time *ΔT* = *T_100_*-*T_0_* is a measure of efficiency for DNA replication. That is, the greater the duplication time *ΔT* is, the less efficient the DNA replication process is at the locus.
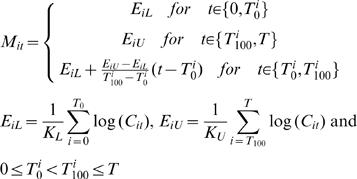
(4)Where *M_it_* is the model of log-ratio of locus *i* at time *t*. All other estimated parameters are for the locus *i* in the notations. *T^i^*_0_ and *T^i^*_100_ are replication initiation timing and completion timing, respectively. *E_iL_* and *E_iU_* are the lower and upper limits of replication, respectively. *K_L_* and *K_U_* are the number of observations for locus *i* up to *T*_0_ and after *T*_100_, respectively. The values are estimated by exhaustive search to minimize the *MSE* (*m*ean-*s*quare *e*rror) between *M_it_* and log(*C_it_*), *i.e.*, 
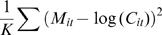
 where *K* is the total number of observations for locus *i* at all time points together. The p-value of the fit was calculated using an ANOVA table. To eliminate errors, the estimates of *T_50_* and *ΔT* were smoothed using a moving average of 3 loci.

#### Prediction of peaks

To predict peaks or potential origins, we applied PeakFinder [25] to identify peaks based on the profiles of replication half-completion timing *T_50_*. We set the parameters of the software as Gaussian Smoothing, Raw data, N = 1, round = 1, Left Delta = Right Delta = 0. The efficiency of origins was approximated using the efficiency of loci at the origins or closest to the origins.

#### Identification of efficient and inefficient replication regions

To identify efficient and inefficient regions, we applied a sliding window to score all the loci by the following equation: 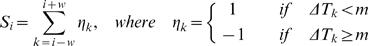
(5)where *m* is the median of *ΔT* at all loci; and *w* is the half window size, so we defined the efficient region in which all loci had a score that was more than the certain threshold *α*; and the inefficient region in which all loci had a score that was less than −*α*. In this study, we set w = 5, *α* = 4, and the minimal number of loci in a region is 19. The median of *ΔT* at all loci is ∼31.6 min in wild type cells and ∼150 min in *cds1Δ* cells.

### Statistical Analysis

A standard binomial test was employed to examine the significant enrichment, and the Wilcoxon rank test was employed to examine the differences between two groups with unknown distribution at various significance levels.

The completed microarray datasets have been submitted to the GEO Databases (the accession number is GSE6977).

## Supporting Information

Table S1 Profiles of replication timing and efficiency in wild type cells
(0.33 MB DOC)

Table S2 Comparisons of peaks/ORI between this study and the studies previously published by others
(0.03 MB DOC)

Table S3 Profiles of replication timing and efficiency in cds1 cells
(0.29 MB DOC)
